# Copper Ions Removal from Water using A_2_B_3_ Type Hyperbranched Poly(amidoamine) Hydrogel Particles

**DOI:** 10.3390/molecules24213866

**Published:** 2019-10-26

**Authors:** Hojung Choi, Youngsik Eom, Sanghwa Lee, Sang Youl Kim

**Affiliations:** Department of Chemistry, Korea Advanced Institute of Science and Technology (KAIST), Daejeon 34141, Korea; ghwnd0214@kaist.ac.kr (H.C.); eomyoungsik@gmail.com (Y.E.); gomeis@kaist.ac.kr (S.L.)

**Keywords:** heavy metal ion removal, poly(amidoamine), hyperbranched polymer, inverse suspension polymerization.

## Abstract

Micrometer-sized hyperbranched poly(amidoamine) (hPAMAM) particles are prepared with a simple A_2_B_3_ type Aza–Michael addition reaction between aminoethylpiperazine (AEP) and methylenebisacrylamide (MBA) in an inverse suspension polymerization condition. The synthesized particles exhibited surprisingly high Cu^2+^ sorption capacity (0.223g/g) for a solid-type absorbent. In addition to the high sorption ability of the particle, its simple synthetic process and convenience, due to its micrometer-sized spherical shape and recyclability, make it a practical and attractive absorbent for heavy metal ion removal from aqueous solutions.

## 1. Introduction

The heavy metal ion pollution of water has been a long-pending problem since the industrial revolution. Although acute heavy metal ion poisoning accidents have disappeared, it is still an ongoing trouble these days. For example, arsenic has been confirmed as a typical toxic metal ion causing abdominal pain and cancer in the past [1], and a high level of arsenic has been found at Ganges, Bangladesh, in 1998 [2], and also in China in 2013 [3]. Another notorious toxic heavy metal ion is mercury. The usage and the disposal of mercury have been curtailed after the disaster in Minamata in 1959, but a high concentration of mercury still exists in several oceans [4] and mercury-accumulated fishes are often found in the oceans [5]. In recent decades, copper has becomes one of the popular technology metals in the industrial field. It is widely used from electrical wires to integrated circuits and thin-film solar cells [6], but copper ions show toxicity to humans, living organisms, and the environment [7]. From this point of view, the removal of the heavy metal ions that are poisoning the aqueous environment is an issue which needs a continuous research effort, in addition to the restriction of the use of toxic heavy metals.

To address the removal of heavy metal ions from water, numerous methods have been suggested, such as precipitation [8], reverse osmosis [9], ion exchange [10,11,12], and adsorption [13]. One of the interesting methods is using poly(amidoamine) (PAMAM) dendrimer as an adsorbent [14,15,16]. Abundant amine functional groups originated from its dendritic nature facilitate heavy metal ion adsorption, resulting in a high adsorption performance. However, PAMAM dendrimer has significant drawbacks. The synthesis of dendrimers is a complicated and time-consuming process to obtain the necessary high molecular weight, and it cannot be separated easily from water, as it needs ultrafiltration because of their nanometer-size hydrodynamic diameters. To overcome these drawbacks several methods, including impregnating/grafting the PAMAM dendrimer onto supports, have been proposed [17,18,19,20,21]. Recently our group suggested a novel method that can overcome the drawbacks of PAMAM dendrimer without supports, utilizing the synthesis of micro-sized gel particles of hyperbranched PAMAM (hPAMAM) [22]. By the slow feeding of one monomer (methylene bisacrylamide, MBA) into the other (ethylenediamine, EDA), highly-branched poly(amidoamine) particles have successfully been obtained in one step. It can be easily separated from water by a micro-size filter and can be produced on a large scale. Interestingly, the synthesized hPAMAM particles show a high sorption capacity of Cu^2+^, at 0.17 g/g.

In this study, we used an A_2_B_3_ type monomer system instead of the previously reported A_2_B_4_ type monomer system [22]. By replacing ethylenediamine (EDA) (a B_4_ type monomer) with 1-(2-aminoethyl)piperazine (AEP) (a B_3_ type monomer), we intended to reduce the crosslinking density while maintaining structural robustness through the cyclic structure together with the tertiary-amine group in the piperazine moiety, which is expected to improve the metal ion binding behavior of hPAMAM hydrogel particles.

## 2. Results and Discussion

**Scheme 1:**
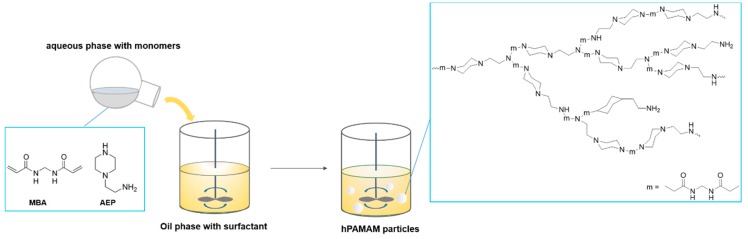
Synthesis of hyperbranched poly(amidoamine) particles from 1-(2-aminoethyl)piperazine (AEP) and N,N′-methylenebisacrylamide.

Aza–Michael addition polymerization between MBA (N,N′-methylenebis acrylamide) and AEP was successfully carried out by following the previously reported method [22]. Because AEP is soluble in water but MBA has a low solubility in water, simple mixing of AEP and MBA in water provides a slow feeding system of MBA into the aqueous solution of AEP. Particle shape can be achieved via inverse suspension polymerization, consisting of water as an aqueous phase and toluene as an organic phase together with span 60 as a water-in-oil (W/O) surfactant. After the polymerization reaches a critical gel point, the aqueous polymer droplets become insoluble gel particles. The polymerization process is depicted in Scheme 1. Synthesized polymers are designated as AEP_x_/MBA_y_/span60_z_, where x and y stand for the monomer feed ratio x:y=[AEP]_0_:[MBA]_0_, and z is the weight concentration (%) of span 60 to monomers, respectively. After preparing AEP/MBA/span60 hPAMAM particles, the synthesized hydrogel particles were characterized.

**Figure 1:**
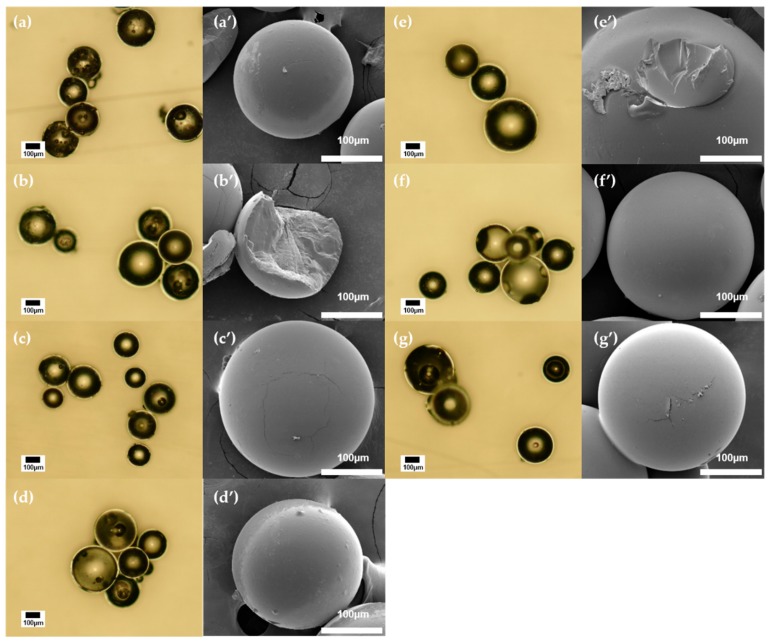
Optical microscopy (OM) images of hyperbranched poly(amidoamine) particles obtained with the following monomer feed ratio; AEP_x_/MBA_1.5_/span60_1_. (a) x = 0.8, (b) x = 0.9, (c) x = 1.0, (d) x = 1.1, (e) x = 1.2, (f) x = 1.3, (g) x = 1.4. Scanning electron microscopy (SEM) images of AEP_x_/MBA_1.5_/span60_1_; (a’) x = 0.8, (b’) x = 0.9, (c’) x = 1.0, (d’) x = 1.1, (e’) x = 1.2, (f’) x = 1.3, (g’) x = 1.4.

In Figure 1, optical microscopy (OM) and scanning electron microscopy (SEM) images of the hPAMAM hydrogel particles obtained with different monomer feed ratios are presented. Since the stoichiometric value of [AEP]:[MBA] is 1:1.5, highly-departed values (under 0.8:1.5 and over 1.4:1.5) cannot induce a gel formation. In all monomer feed ratios employed, the polymer particles were obtained with a spherical shape and size of 50–300 micrometers.

**Figure 2:**
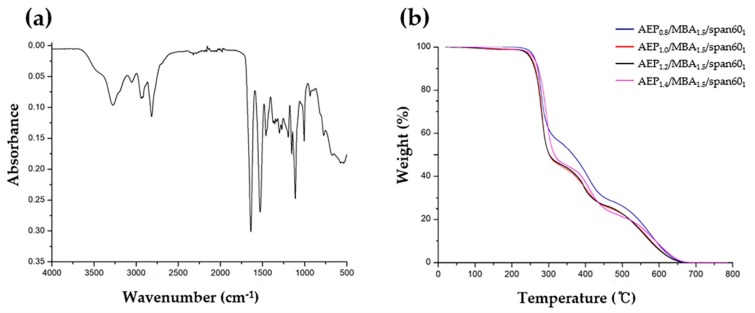
Attenuated total reflection infrared spectroscopy (ATR-IR) (**a**), and thermal gravimetric analysis (TGA) data (**b**). The ATR-IR sample was AEP_1.2_/MBA_1.5_/span60_1_ in (**a**).

The chemical structure of the polymers was characterized by attenuated total reflection infrared spectroscopy (ATR-IR) spectroscopy (Figure 2a). Amine N-H stretch (3300~3400 cm^−1^), C-H stretch (2820 cm^−1^), and amide C=O (1640 cm^−1^) signals clearly indicate that polymerization was carried out successfully. The signal of 3060 cm^−1^ is assigned as an overtone of the 1530 cm^−1^ signal, often found in amide compounds, and not the C=C double bond signal from the unreacted MBA. For further verification, proton nuclear magnetic resonance (^1^H-NMR) spectroscopic analysis of the polymer obtained with an AEP MBA 1.2: 1.5 monomer feed ratio was carried out, and the spectrum showed no C=C double bond peak, while the polymer still showed the 3060 cm^−1^ signal in ATR-IR (Figure S5). Representative thermal gravimetric analysis data are presented in Figure 2b. All polymers showed good thermal stability for nitrogen-containing aliphatic polymers. The temperature of 5% weight loss (T_d,5%_) was 266 °C on average. This high thermal stability of the hPAMAM particles implies that the polymer network is highly stable without supports such as silica.

The sorption feature of the hyperbranched PAMAM polymers was studied. In general, swelling of a cm-scale hydrogel is presented by volume or length change through swelling. However, synthesized particles were in the range of 50–300 μm, which is quite small compared to macroscopic gels. Therefore, we examined their swelling behavior with the weight change instead of length change. The water swelling ratio of the particles was obtained via the beaker test method with distilled water for 24 h. Because the water uptake was mainly affected by the crosslink density of the polymer hydrogel, the swelling ratio of particles increased with increasing deviation from its stoichiometric value, [AEP]:[MBA]=1:1.5 (detailed information are presented in Figure S1). The maximum swelling ratio was 895% in AEP_1.4_/MBA_1.5_/span60_1_, and the minimum swelling ratio was 335% in AEP_1_/MBA_1.5_/span60_1._

Subsequently, the Cu^2+^ absorption capacity was measured with an inductively coupled plasma optical emission spectrometer (ICP-OES), by following the concentration change of the CuCl_2_ solution (1000 ppm) after placing hPAMAM particles in the solution for a certain time. Copper absorption capacities exhibited an expected tendency, proportional to the increase of AEP units. The highest Cu^2+^ sorption capacity of 0.223 g/g was observed with AEP_1.4_/MBA_1.5_/span60_1_, suggesting that the amine functionalities are strongly related to the Cu^2+^ sorption ability. Noteworthy, 0.223 g/g is quite a high value for a solid-type absorbent and almost quadruple to that of commercial resin, Dowex M4195 (Table 1). In addition, the highest Cu^2+^ absorption capacity of the AEP-MBA gel is 31% higher than the value of the EDA-MBA hPAMAM particles, which was previously reported by our group [22].

**Figure 3:**
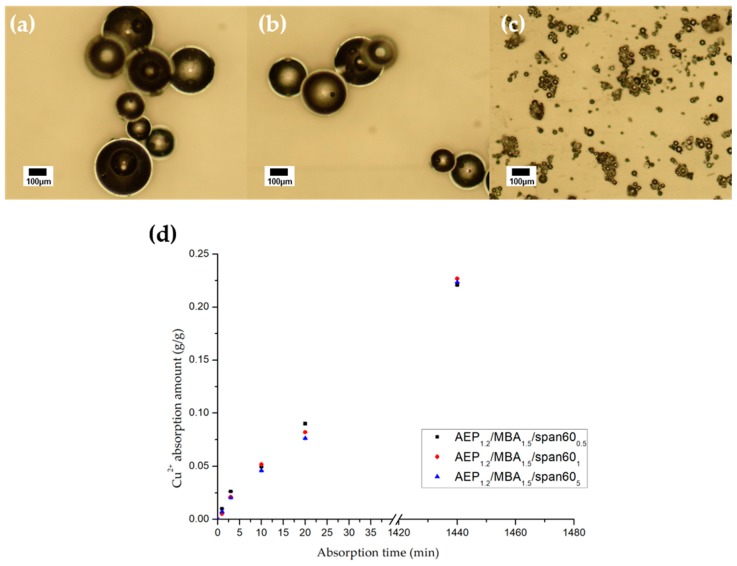
Size change of the hPAMAM particles with different span60 concentration. OM images of AEP_1.2_/MBA_1.5_/span60_0.5_ (**a**), AEP_1.2_/MBA_1.5_/span60_1_ (**b**), AEP_1.2_/MBA_1.5_/span60_5_ (**c**), graph of copper (II) ion absorption amount at 1, 3, 10, 20 min, and 24 h in the three surfactant concentrations (**d**).

Next, we studied the time-dependent Cu^2+^ sorption behavior to get information about the sorption rate. Especially, we measured the sorption rate in different particle sizes to understand the heavy metal ion sorption mechanism of hyperbranched PAMAM hydrogel particles (Figure 3). Because the “absorption” process occurs through the whole volume of the particle and not just on the surfaces and “adsorption” occurs on the surfaces, the sorption rate and capacity of the particles with different diameters should provide useful information of its sorption mechanism. The sorption rate of AEP_1.2_/MBA_1.5_/span60_0.5_ (Figure 3a, 100–350 nm), AEP_1.2_/MBA_1.5_/span60_1_ (Figure 3b, 100–300 nm), and AEP_1.2_/MBA_1.5_/span60_5_ (Figure 3c, 20–80 nm) in early stages showed none or negligible differences, although they have a large difference of particle diameters. Additionally, the sorption capacities of three particles showed similar values of about 0.205 g/g (Figure S2), indicating that the surface area is not a crucial factor, and that absorption is the major sorption mechanism of the hPAMAM hydrogel particles. Moreover, the existence of copper inside of the particle has been confirmed by energy dispersive X-ray spectroscopy (EDX) analysis on a cross-section of the Cu^2+^-absorbed particle, which supports the absorption mechanism (Table S2).

Another important factor for heavy metal sorbent is desorption. Some strong sorbents showing high sorption capacities have low desorption, requiring additional desorbents such as ethylenediaminetetraacetic acid(EDTA) [30,31,32,33]. These additional treatment processes limit the usability and applicability of sorbents. To check the desorption ability of synthesized hPAMAM particles, Cu^2+^ saturated hPAMAM hydrogel particles were treated with 0.1N HCl aqueous solution for 3 h, and then energy dispersive X-ray spectroscopy (EDX) analysis was carried out. It was found that the acid treated PAMAM showed zero copper contents in thye EDX elemental analysis, meaning that complete desorption was made (Figure S4, Table S1).

## 3. Materials and Methods

### 3.1. Materials

1-(2-Aminoethyl)piperazine (AEP), N,N′-Methylenebisacrylamide (MBA), Span 60^®^, and CuCl_2_ anhydrous were purchased from Sigma-Aldrich (St. Louis, MI, USA). Toluene was purchased from Junsei (Japan). All the reactants were used without further purification. Optical microscopy (OM) images were obtained on a Nikon Eclipse ME600 (Nikon, Tokyo, Japan), and field emission scanning electron microscopy (FE-SEM) images were obtained using a FEI Company Inspect F50 (USA) and a Hitachi SU 8230 (Japan). Energy-dispersive X-ray analysis (EDX) was performed with a Hithchi SU 8230 (Hithchi, Tokyo, Japan). Metal ion concentration was measured by an inductively coupled plasma optical emission spectrometer (Agilent ICP-OES 720, Santa Clara, CA, USA). Fourier transform infrared spectroscopy (FT-IR) spectra were obtained from a Nicolet iS50 (Thermo Fisher Scientific, Waltham, MA, USA), and thermogravimetric analysis (TGA) was performed on a TGA Q50 (TA Instruments, New Castle, DE, USA) in N_2_ condition.

### 3.2. Methods

#### 3.2.1. Synthesis of Poly(Amidoamine) Particles

Inverse suspension polymerization was applied to synthesize the polymer particles. Oil phase was prepared in a 50 mL round bottom flask as the suspension stabilizer span 60 (1.0 wt% of the monomers) was dissolved in cyclohexane (12 mL) and heated to 60 °C with vigorous agitation. Methylenebis(acrylamide) (MBA) was placed in a 50 mL round bottom flask with water (6 mL) and heated to 50 °C. AEP was added into the MBA solution. The aqueous mixture was heated at 50 °C for 5–10 min until the two monomers completely dissolved. After the aqueous solution became transparent, it was poured into the oil phase solution and then agitated in 1000 rpm at 60 °C for 12 h. After polymerization, off-white polymer hydrogel particles were produced and these particles were filtered and washed several times with distilled water, acetone and methanol.

#### 3.2.2. Measurement of Copper Ion Absorption Capacity

Stock copper solution was prepared with the initial concentration of copper (C_i_) in deionized water. Dry polymer particles were placed in a 20 mL vial with a certain volume of the stock copper solution (V) and kept for 24 h to reach sorption saturation at room temperature. Copper absorbed polymer particles were removed by 0.45 μm Nylon syringe filter, and the filtrate were collected. The concentration of copper in the filtrate (C_f_) was determined by inductively coupled plasma optical emission spectrometry (ICP-OES). Copper ion absorption capacity (A) was calculated from the following equation, Equation (1).

Copper ion absorption capacity (A) = [(C_i_ − C_f_)V/m] (g/g) (1)

where m (mg) is the weight of the dry sample, and V (mL) is the volume of the stock copper solution. C_i_ and C_f_ (mg/mL) are the initial and filtrate copper ion concentrations, respectively. The sorption rate is defined as the copper sorption amount (g/g) divided by sorption time (min).

## 4. Conclusions

In conclusion, micro-sized hPAMAM hydrogel particles consisting of AEP and MBA were successfully synthesized by a simple A_2_B_3_ type Aza–Michael addition, via inverse suspension polymerization. The synthesized particles showed a high absorption capacity of Cu^2+^ regardless of its diameter, as high as 0.223 g/g, which is 31% higher than ethylenediamine-based hPAMAM particles and 412% of the commercial sorbent Dowex M4195. High sorption capacity, easy desorption at mild conditions, and handy micrometer-sized particle diameters make these hydrogel particles useful materials for a practically efficient absorption system e.g., a packed column. Amine and amido groups can bind not only copper but also other metals, e.g., cadmium. Further investigation on this proposed polymer sorbent can be expanded, for its use as a high-performance sorbent for various heavy metal ions.

## Figures and Tables

**Table 1:** Copper (II) ion sorption capacities of various sorbents. hPAMAM: Hyperbranched poly(amidoamine); EDA: Ethylenediamine.

Sorbent	Cu^2+^ Sorption Capacity (g/g)	Reference
Dowex M4195	0.054	[22]
Graphene oxide/Fe_3_O_4_	0.023	[23]
PEI-PS resin	0.116	[24]
m-MCM-41/PMMA	0.042	[25]
m-KIT-6	0.102	[26]
Alginate@PEI-1.5	0.164	[27]
Polyaniline grafted chitosan beads	0.100	[28]
Magnetic chitosan beads	0.147	[29]
PAMAM dendrimer	0.329	[15]
EDA-MBA hPAMAM particles	0.170	[22]
AEP-MBA hPAMAM particles	0.223	This work

## Supplementary Materials

The following are available online at https://www.mdpi.com/1420-3049/24/21/3866/s1, Figure S1. Water swelling ratio of AEP_x_/MBA_1.5_/span60_1_, Figure S2. Cu2+ absorption for different surfactant conditions (z = 0.5, 1, 2, 5 in AEP_1.2_/MBA_1.5_/span60_z_, absorption time was 24h), Figure S3. Cu2+ absorption saturation test (used polymer: AEP_1.2_/MBA_1.5_/span60_1_), Figure S4. Images of AEP_1.2_/MBA_1.5_/span60_1_ in absorption and desorption process, Figure S5. 1H-NMR and IR spectra of AEP_1.2_/MBA_1.5_ polymer, Table S1. Elemental analysis of Copper by EDX spectroscopy, Table S2. Elemental analysis of Copper on surface and cross-section of the particles by EDX.
