# Supplementary material for: Copper Ions Removal from Water using A2B3 Type Hyperbranched Poly(amidoamine) Hydrogel Particles
Source: Molecules. 2019 Oct 26;24(21):3866. doi: 10.3390/molecules24213866 (PMC6864665; doi:10.3390/molecules24213866)
Supplement: Supplementary file 1 [file molecules-24-03866-s001.pdf]

Supplementary Materials

Communication

Copper Ions Removal from Water using A_2B_3 Type Hyperbranched Poly(amidoamine) Hydrogel Particles

Hojung Choi, Youngsik Eom, Sanghwa Lee and Sang Youl Kim*

[1] Department of Chemistry, Korea Advanced Institute of Science and Technology (KAIST), Daejeon, 34141, Korea; ghwnd0214@kaist.ac.kr (H.C.); eomyoungsik@gmail.com (Y.E.); gomeis@kaist.ac.kr (S.L.)

* Correspondence: kimsy@kaist.ac.kr; Tel.: +82-42-350-2834

Academic Editor: Encarnación Ruiz Ramos

Received: 28 September 2019; Accepted: 24 October 2019; Published: 26 October 2019

Measurement of water swelling ratio

Dried polymer particles (W_d) were placed in distilled water and kept for 24 hours to achieve full equilibrium at room temperature. Swollen polymer particles were filtered to remove remaining water on the particle surface. Then the polymer particles were collected and weighed (W_s). Swelling ratio (Q) was calculated from the following equation, Equation (1).

$$\text{Swelling ratio (Q)} = [(W_s-W_d)/W_d * 100]\ (\%) \qquad (1)$$

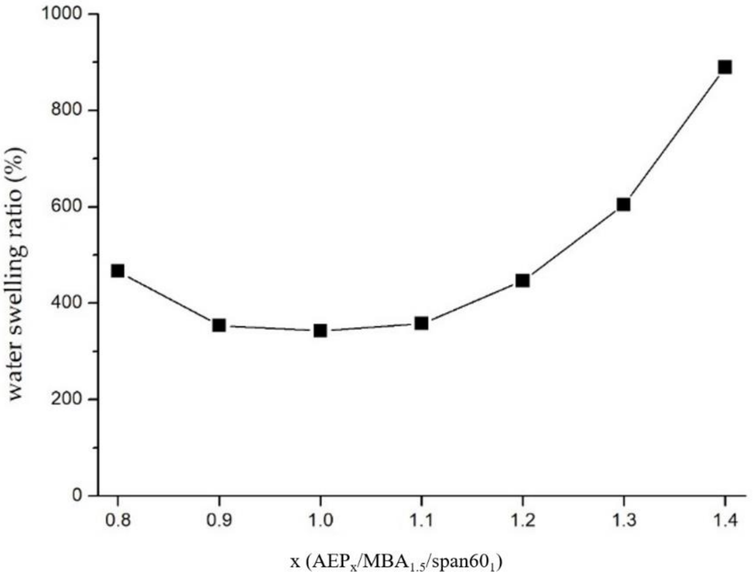

Figure S1. Water swelling ratio of AEP$_x$/MBA$_{1.5}$/span60$_1$**.**

Complementary Cu2+ Absorption Tests

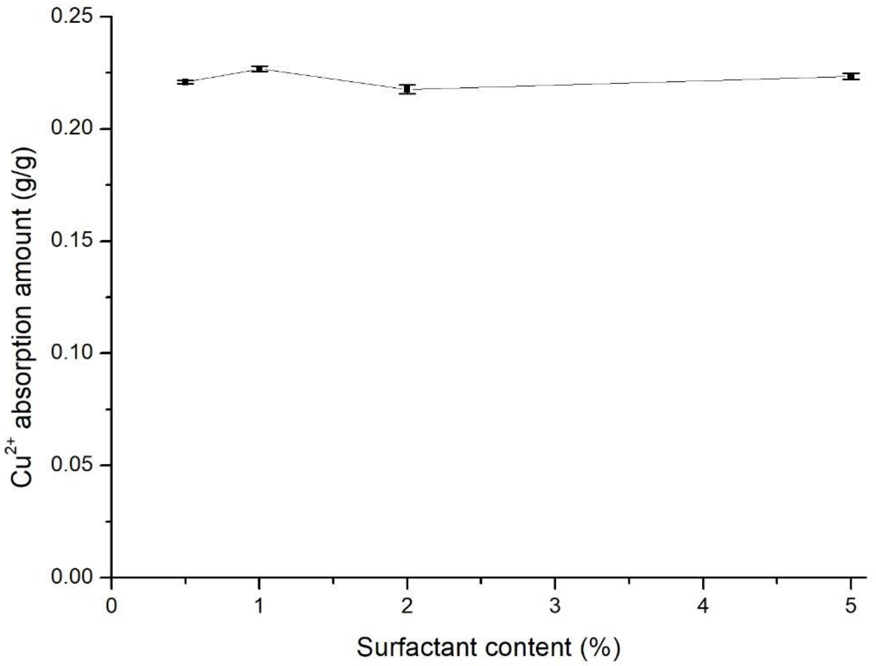

Figure S2. Cu2+ absorption for different surfactant conditions (z = 0.5, 1, 2, 5 in $AEP_{1.2}/MBA_{1.5}/span60_z$, absorption time was 24h).

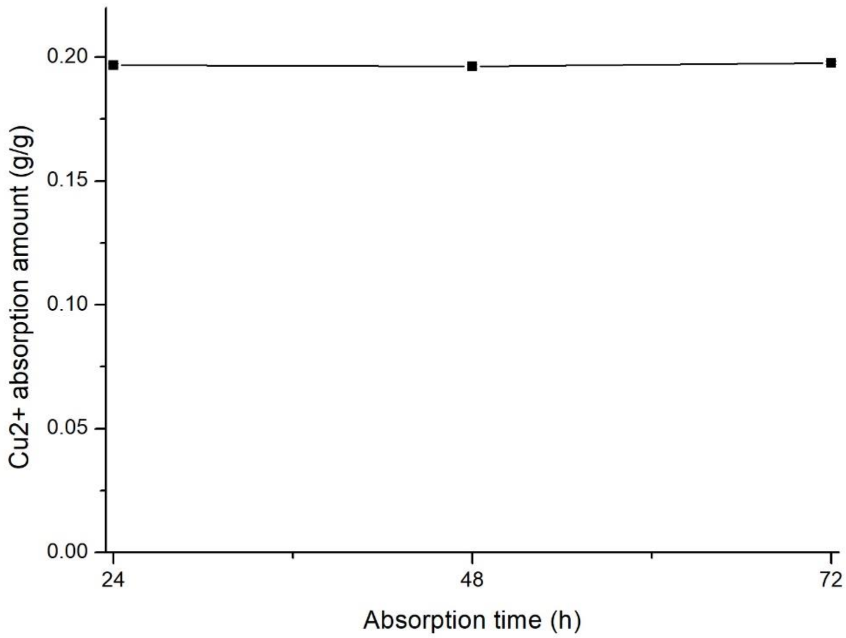

Figure S3. Cu2+ absorption saturation test (used polymer: $AEP_{1.2}/MBA_{1.5}/span60_1$).

Measurement of Copper (II)Ion Sorption/Desorption by EDX analysis

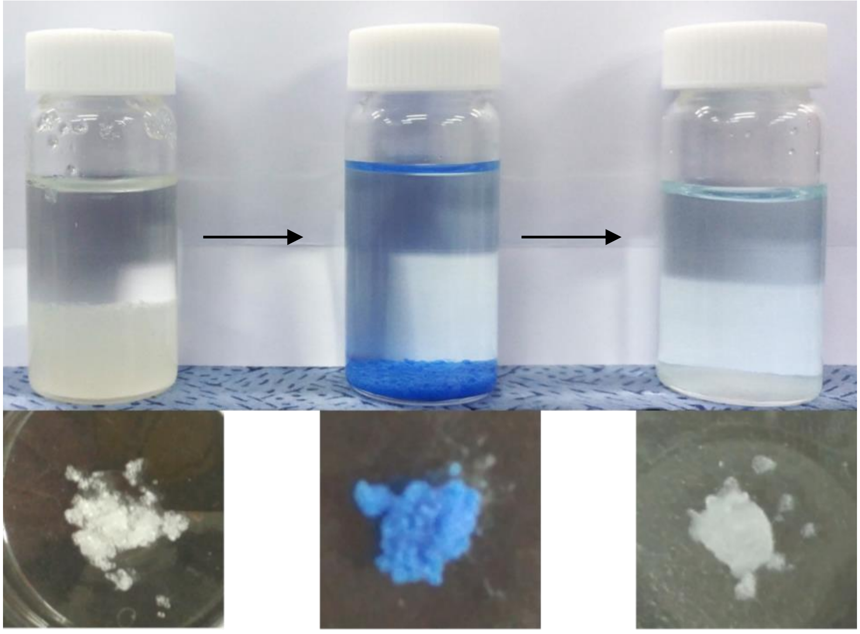

Synthesized **Cu^{2+} absorbed** **Acid treated**

Figure S4. Images of $AEP_{1.2}/MBA_{1.5}/span60_{1}$ in absorption and desorption process.

Table S1. Elemental analysis of Copper by EDX spectroscopy.

	Weight (%)		
Element	Synthesized PAMAM	Cu (II) adsorbed PAMAM	Acid treated Cu (II) adsorbed PAMAM
Cu	0.00	36.94	0.00

Element composition was measured by EDX instrument on Hitachi SU8230.

Table S2. Elemental analysis of Copper on surface and cross-section of the particles by EDX.

	Weight (%)	
Element	Surface of PAMAM	Cross-section of PAMAM
Cu	45.46	52.08

Element composition was measured by the EDX instrument Hitachi SU8230.

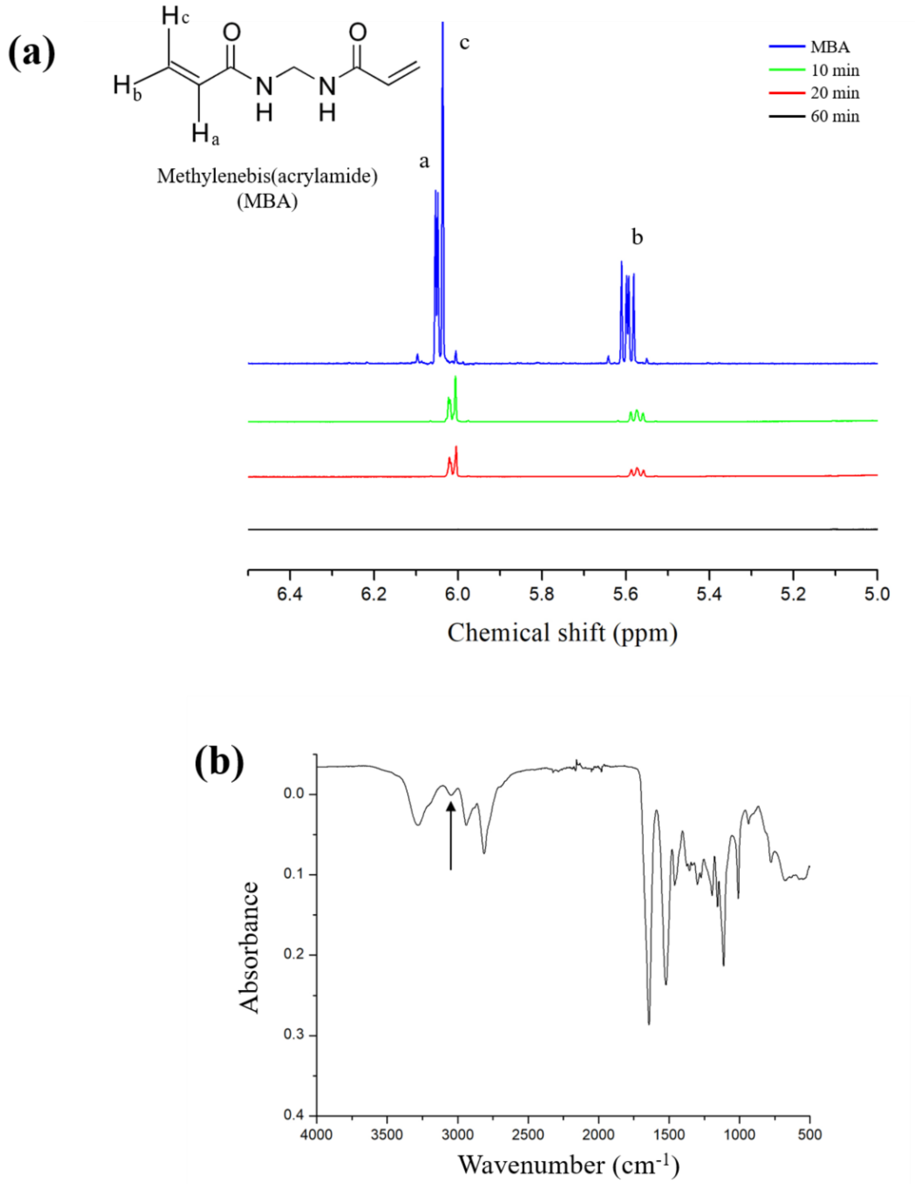

Figure S5. 1H-NMR and IR spectra of $AEP_{1.2}/MBA_{1.5}$ polymer.

To show that the band of 3060 cm^{-1} in ATR-IR data is not originated from the unreacted C=C double bond, polymerization of $AEP_{1.2}/MBA_{1.5}/span60_{0}$ bulk hydrogel was monitored over time by 1H-NMR. (Figure S3a) Before gelation (~1h), the polymer solution was diluted in D_2O. Between 20 minutes and 60 minutes after polymerization begins, the C=C double bond disappeared. After six hours of polymerization, synthesized $AEP_{1.2}/MBA_{1.5}/span60_{0}$ bulk hydrogel was crushed with a spatula and the ATR-IR spectrum was taken. (Figure S3b) 3060 cm^{-1} band (marked with arrow) also appeared in the sample which has no C=C double bond confirmed by 1H-NMR, providing an evidence that the band is an overtone of 1530cm^{-1} often found in alkyl acetamides e.g. N-ethylacetamide, [1] N-(n-propyl)acetamide [2].

Reference

1. NIST Chemistry WebBook. Available online: https://webbook.nist.gov/cgi/cbook.cgi?ID=C5331486&Mask=80 (accessed on 25 09 2019).
2. Nist Chemistry WebBook. Available online: https://webbook.nist.gov/cgi/inchi?ID=C625503&Type=IR-SPEC&Index=1 (accessed on 25 09 2019)
